# Constrictive Pericarditis in the Presence of Remaining Remnants of a Left Ventricular Assist Device in a Heart Transplanted Patient

**DOI:** 10.1155/2015/372698

**Published:** 2015-05-21

**Authors:** R. Rivinius, M. Helmschrott, V. Koch, F. Sedaghat-Hamedani, P. Fortner, F. F. Darche, D. Thomas, A. Ruhparwar, B. Schmack, M. Karck, M. Akhavanpoor, C. Erbel, C. A. Gleissner, S. J. Buss, D. Mereles, P. Ehlermann, H. A. Katus, A. O. Doesch

**Affiliations:** ^1^Department of Cardiology, Angiology and Pneumology, University of Heidelberg, 69120 Heidelberg, Germany; ^2^Department of Cardiac Surgery, University of Heidelberg, 69120 Heidelberg, Germany

## Abstract

Constrictive pericarditis (CP) is a severe subform of pericarditis with various causes and clinical findings. Here, we present the unique case of CP in the presence of remaining remnants of a left ventricular assist device (LVAD) in a heart transplanted patient. A 63-year-old man presented at the Heidelberg Heart Center outpatient clinic with progressive dyspnea, fatigue, and loss of physical capacity. Heart transplantation (HTX) was performed at another heart center four years ago and postoperative clinical course was unremarkable so far. Pharmacological cardiac magnetic resonance imaging (MRI) stress test was performed to exclude coronary ischemia. The test was negative but, accidentally, a foreign body located in the epicardial adipose tissue was found. The foreign body was identified as the inflow pump connection of an LVAD which was left behind after HTX. Echocardiography and cardiac catheterization confirmed the diagnosis of CP. Surgical removal was performed and the epicardial tubular structure with a diameter of 30 mm was carefully removed accompanied by pericardiectomy. No postoperative complications occurred and the patient recovered uneventfully with a rapid improvement of symptoms. On follow-up 3 and 6 months later, the patient reported about a stable clinical course with improved physical capacity and absence of dyspnea.

## 1. Introduction

Pericardial disorders have been described as common complications after heart transplantation (HTX) [1]. Mostly benign, treated with medication, and self-limiting, pericarditis can be complicated by constriction increasing morbidity and mortality [2]. This severe subform of pericarditis is named constrictive pericarditis (CP) and has a reported incidence of 1.4% to 3.9% in patients after HTX [3, 4]. It is characterized by an inflamed, fibrotic, and thickened pericardium limiting the ventricle's distensibility and therefore resulting in a reduced venous return with impaired diastolic ventricular function [5].

Various causes for CP exist, most often idiopathic [2]. In patients with identified etiology, prior cardiac surgery is the main cause for CP [6, 7]. Signs and symptoms of CP share a common ground with multiple entities. Once focus is set on CP, it remains a difficult challenge even for skilled clinicians to distinguish between CP and restrictive cardiomyopathy as both share similar clinical features [8].

Here, we present the unique case of CP in the presence of remaining remnants of a left ventricular assist device (LVAD) in a heart transplanted patient.

## 2. Case Presentation

A 63-year-old man presented at the Heidelberg Heart Center outpatient clinic with progressive dyspnea, fatigue, and loss of physical capacity. Chest pain, palpitations, or syncope was denied. HTX was performed at another heart center four years ago and postoperative clinical course was unremarkable so far. Last coronary angiography had been without evidence of coronary artery disease and left ventricular ejection fraction had not been impaired. There had been no cellular or humoral rejection episodes.

**Figure 1:**
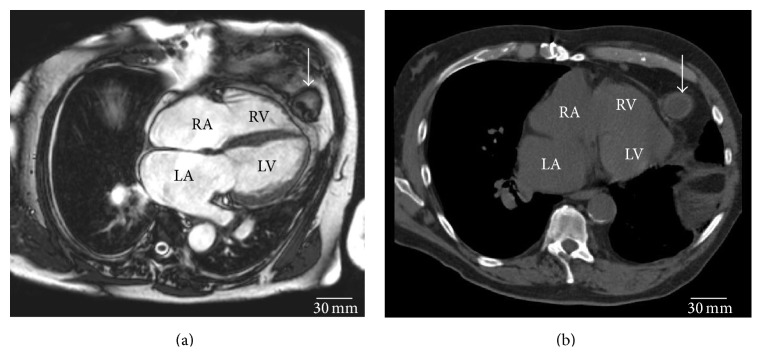
Epicardial foreign body (arrow) with a diameter of 30 mm in (a) cardiac magnetic resonance imaging (MRI) and (b) thoracic computed tomography (CT): diffuse thickening of the pericardium surrounding the foreign body with pericardial effusion is shown. RA denotes right atrium, LA left atrium, RV right ventricle, and LV left ventricle.

Resting electrocardiography (ECG) showed a sinus rhythm at a rate of 87/min without further abnormalities. Current echocardiography showed a normal size and systolic function of both ventricles with enlarged atria. Pharmacological cardiac magnetic resonance imaging (MRI) stress test was performed to exclude coronary ischemia. The test was negative but, accidentally, a foreign body located in the epicardial adipose tissue was found (Figure 1(a)). For further assessment, thoracic computed tomography (CT) was performed (Figure 1(b)).

The foreign body was identified as the inflow pump connection of the LVAD which was left behind after HTX. Upon request at the external heart center where the transplantation was performed four years ago, the information was given that the inflow pump connection of the LVAD remained in situ after HTX due to severe adhesions.

Meanwhile, the patient's general health condition worsened. Cardiac auscultation exposed a pericardial friction rub along the left sternal border without other valvular murmurs. Pulmonary status indicated bilateral basal crackles and a left-sided muffled sound with dullness to percussion. Also, elevated jugular venous pressure on inspiration (Kussmaul's sign) and pitting edema in the lower extremities as well as an extended abdomen could be observed.

**Figure 2:**
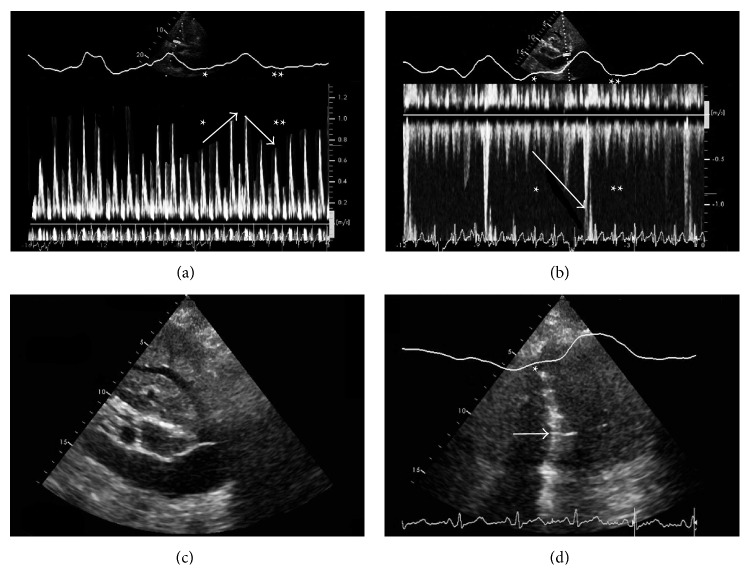
Echocardiographic features of constrictive pericarditis. (a) Pulse wave Doppler recording of tricuspid inflow in apical four-chamber view: increased early diastolic filling velocity during inspiration (ascending arrow), opposite during expiration (descending arrow). (b) Pulse wave Doppler recording of hepatic vein inflow in subcostal view: increased velocity during inspiration (descending arrow). (c) Dilated inferior vena cava and hepatic veins with restricted respiratory variation in subcostal view. (d) Abnormal interventricular septum motion (septal bounce) as a sign of ventricular interdependence in apical four-chamber view: septal bounce to the left ventricle during inspiration (arrow).

Follow-up echocardiography showed an increased echogenicity in the region of the pericardium (pericardial thickness ≥ 3 mm). During inspiration, an abnormal interventricular septal bounce to the left was found whereas the opposite was observed during expiration (ventricular interdependence). Additionally, distinct paradoxical right and left diastolic ventricular filling were seen depending on the respiratory system pressure gradient. Also, there was a dilatation of the inferior vena cava and hepatic veins with diminished respiratory variation. Echocardiographic findings are summarized in Figure 2.

**Figure 3:**
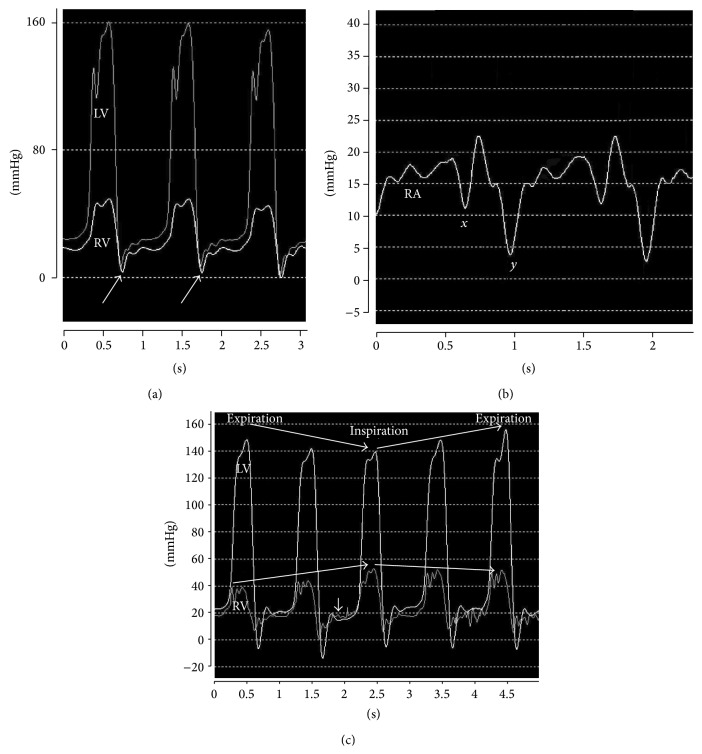
Cardiac catheterization findings of constrictive pericarditis. (a) Ventricular pressure tracings indicating a characteristic dip-and-plateau waveform (square root sign) (arrow). (b) Right atrial pressure tracing showing a prominent *x*- and *y*-descent (W sign). (c) Changes of ventricular pressure during respiration: increased right ventricular pressure with concordant decreased left ventricular pressure during inspiration. The opposite is found during expiration. Further, end-diastolic equalization of pressures in both ventricles is observed (arrow). RA denotes right atrium, RV right ventricle, and LV left ventricle.

Right- and left-sided cardiac catheterization were performed to confirm the diagnosis of CP and to exclude differential diagnoses. Here, hemodynamic criteria for CP including diastolic dysfunction were recorded. Ventricular pressure tracings revealed a characteristic dip-and-plateau waveform consisting of a pronounced dip in early diastolic filling with a subsequent rapid rise with reduced filling in mid-diastole characterized by a plateau (square root sign). Late diastolic filling exposed elevated right and left ventricular pressure with a right ventricular end-diastolic pressure greater than one-third of the right ventricular systolic pressure. Additionally, right atrial pressure tracing showed a prominent *x*- and *y*-descent (W sign) (Figure 3).

The case was discussed in an interdisciplinary board conference for concerted action regarding the foreign body. As a result, surgical removal was recommended and consequently performed. The epicardial tubular structure with a diameter of 30 mm was carefully removed accompanied by pericardiectomy. No postoperative complications occurred and the patient recovered uneventfully with a rapid improvement of symptoms.

On follow-up 3 and 6 months later, the patient reported about a stable clinical course with improved physical capacity and absence of dyspnea.

## 3. Discussion

The pathophysiology of CP is characterized by inflamed, fibrotic, and thickened pericardial linings resulting in pericardial constriction and impaired ventricular extension [5]. Particularly diastolic function is impeded due to limited cardiac venous return, reduced ventricular filling, and the inability to achieve sufficient preload (Frank-Starling law), whereas the systolic function is seldom affected [9].

Multiple causes for CP are known including infections (viral, fungal, tubercular, or parasitic), inflammatory or autoimmune diseases, mediastinal radiation therapy, or neoplastic pericardial infiltration, though most cases remain idiopathic [2]. There exist occult, subclinical, or fulminant forms which can emerge slowly or rapidly. The three main causes for CP are idiopathic etiology (presumably viral), cardiac surgery, and radiation therapy [6].

Signs and symptoms of CP can include dyspnea, fatigue, loss of physical capacity, tachycardia, fever, pitting edema in the lower extremities, and abdominal swelling [2]. Additionally, Kussmaul's sign and pulsus paradoxus can be observed as signs of impaired ventricular diastolic filling [7].

Diagnostic procedures comprise ECG, cardiac catheterization, thoracic CT, and cardiac MRI [4, 10]. Most common echocardiographic findings in CP are an impaired diastolic ventricular filling with enlarged atria due to elevated atrial pressure and a preserved systolic ventricular function. Typically, a paradoxical interventricular septum motion (septal bounce) reflecting the ventricular interdependence is observed [11].

Cardiac catheterization can be used to distinguish between CP and restrictive cardiomyopathy. Both entities share an early accelerated filling followed by a rapid elevation and an end-equalization of ventricular pressures (square root sign) [8]. In CP, there is an increased right ventricular pressure with concordant decreased left ventricular pressure during inspiration whereas the opposite is found during expiration [12]. In contrast hereto, restrictive cardiomyopathy shows a simultaneous decrease of right and left ventricular pressure during inspiration and a simultaneous increase during expiration [9].

To determine pericardial thickness, inflammatory reaction, fibrotic organization, pericardial effusion, or the degree of calcification, CT or MRI may be applied [4, 10].

General treatment for CP is pericardiectomy providing a curative approach [13, 14]. Long-term survival after pericardiectomy for CP is related to the underlying cause and the clinical condition of the patient. The relatively good survival with idiopathic CP emphasizes the safety of pericardiectomy [13, 14].

Infection, acute rejection, and cardiac allograft vasculopathy have been described as major risk factors for patients with HTX [12]. During the last decades, several reports about CP after HTX have been published [1, 3, 4, 10, 12, 15–18]. In those, time from HTX until diagnosis of CP ranges from 3 weeks up to 11 years and incidence of CP in patients after HTX varies from 1.4% (4 of 295) to 3.9% (5 of 127) [3, 4].

The earliest case report of CP after HTX was published in 1986 and depicts a 37-year-old man who was transplanted due to idiopathic cardiomyopathy. His postoperative course was complicated by numerous infectious complications and repeated open-chest surgery including pericardiectomy for CP [15]. In a study with 133 HTX recipients from 1994, 2 patients developed CP [1]. Both patients had initially a localized pericardial hematoma which may have been the cause for pericardial thickening and constriction [1]. Two further case reports from 1995 and 2005 indicated an association between posttransplant wound infection/mediastinitis and CP [17, 18]. In a case series of 5 patients with CP from 2010, all 5 patients had pericardial effusion of noninfectious etiology in the early posttransplant period [4]. These case reports are in line with the hypothesis that postoperative CP is a result of inflamed, fibrotic, and injured pericardial linings building up an adhesion formation [19].

In HTX recipients, the development of CP may also be related to allograft rejection. A case series with 4 patients from 1994 displays the potential association of allograft rejection with the development of CP [3].

The combination of both, pericardial effusion and rejection episode, was seen in one case of CP after HTX in 2008 [10].

Therefore, the development of CP after HTX seems to be more likely in patients with pericardial effusions and in patients with rejection episodes. Interestingly, our patient neither had pericardial effusion, wound infection, or mediastinitis after HTX nor had any major rejection episode. It might be possible that the remaining remnants of the LVAD located in the epicardial adipose tissue have increased the risk of developing CP by causing chronic subliminal irritation to the pericardium.

In conclusion, CP is a severe subform of pericarditis with various causes and clinical findings. It has to be taken into consideration as a differential diagnosis in symptomatic patients after HTX with preserved systolic ventricular function. Prompt recognition of the underlying cause, exclusion of other etiologies such as restrictive cardiomyopathy, and adequate treatment are essential.

We here present the unique case of CP in the presence of a foreign body located in the epicardial adipose tissue in a heart transplanted patient. The foreign body was identified as the inflow pump connection of the LVAD which remained in situ after HTX due to severe adhesions. After extensive surgery, the remaining remnants of the LVAD could be removed and the patient recovered quickly.
